# The effect of IL-1β inhibitor canakinumab (Ilaris®) on IL-6 production in human skeletal muscle cells

**DOI:** 10.1371/journal.pone.0316110

**Published:** 2025-03-06

**Authors:** Anna Cordeiro-Santanach, Fiorella Morales, Maria del Carmen Parquet, Kitipong Uaesoontrachoon, Joyce Rowsell, Jordan Warford, Wilson Wu, Pia Elustondo, Eric P. Hoffman, Kanneboyina Nagaraju

**Affiliations:** 1 AGADA Biosciences Inc., Halifax, Nova Scotia, Canada; 2 School of Pharmacy and Pharmaceutical Sciences, Binghamton University, State University of New York, New York, United States of America; University of Melbourne, AUSTRALIA

## Abstract

Muscle inflammation is one of the hallmarks of Duchenne muscular dystrophy (DMD). Dystrophin-deficient skeletal muscle cells produce higher levels of pro-inflammatory cytokines such as interleukin 1β (IL-1β) in response to toll-like receptor stimulation compared to normal muscle skeletal cells. IL- 1β induces the human skeletal muscle secretion of the myokine Interleukin-6 (IL-6). Here, we evaluated the effect of a human IgG1κ monoclonal antibody (canakinumab (Ilaris®)) that specifically blocks the IL-1β effect on IL-6 secretion by human skeletal muscle cells. Canakinumab is an excellent candidate for therapeutic repositioning to treat DMD because it is an FDA-approved drug to treat periodic fever syndromes and systemic juvenile idiopathic arthritis. Unlike previous generations of IL-1 inhibitors, canakinumab is highly specific for the IL-1β ligand, has a longer half-life, and does not interfere with other IL-1-activated inflammatory pathways. Following cell culture optimization and viability assays to assess toxicity, skeletal muscle cells were stimulated with IL-1β (10 ng/mL) for 48 hours in the presence of nine concentrations of canakinumab ranging from 0.001 nM to 1000 nM, and IL-6 production was measured with an enzyme-linked immunosorbent assay. Pre-incubation of myoblasts with canakinumab before IL-1β-stimulation, significantly reduced IL-6 production at concentrations of 1, 10, 100, 250, and 1000 nM relative to controls, yielding an IC_50_ of 0.264 nM. On the other hand, co-incubation of canakinumab with IL-1β before addition to myoblasts resulted in a significant inhibition with the IC_50_ reducing to 0.126 nM, less than half of the previous method. Canakinumab also did not affect myotube viability at 10 nM and was also able to significantly reduce the production of IL-6, when the cells were stimulated with IL-1β (10 ng/ml). Taken together, our results show that canakinumab is a potent inhibitor of IL-1β signaling in muscle cells. These results align with previously published pre-clinical work with other IL-1 inhibitors in the mdx mouse model and support further investigation into the clinical utility of repositioning canakinumab to treat DMD.

## Introduction

Duchenne muscular dystrophy (DMD) is a recessive X-linked severe neuromuscular disorder caused by mutations in the dystrophin gene that affects 1:5000 male births. This disease, diagnosed during early childhood, is characterized by progressive muscle weakness, leading to cardiorespiratory failure when the patients are in their mid-20s. There is currently no cure for DMD, and all efforts are targeted to alleviate the symptoms and/or to prolong ambulation [1].

Inflammation is part of the natural process of healing the muscle tissue after trauma. The leakage of the cytoplasm of the affected cells triggers the innate immune response, which supports the healing of the broken cell membrane and resolves the inflammation [2]. Chronic inflammation in muscular tissue is one of the hallmarks of the DMD. Exacerbation of the inflammation causes cycles of necrosis and regeneration of the dystrophin-deficient fibers, decreasing the muscle mass and leading to the loss of muscle function [3]. Dystrophin plays a role in stabilizing the cytoskeleton of myofibers [4]. When myofibers are dystrophin-deficient, the cellular membrane breaks during contraction and fails to repair which affects negatively the performance of the muscle, activating pathways leading to inflammation, fibrosis and necrosis [5–7] and failed regeneration of muscle cells [8]. Recent studies from our group demonstrated that normal regeneration and failed regeneration involve the same gene networks, differences largely being caused by the synchronic (episodic) nature of normal regeneration and the asynchronous (repeated injury) nature of failed regeneration. With failed regeneration, regenerating myofibers receive incorrect temporal cues from their neighbors, and this inappropriate crosstalk leads to the inability to progress through the normal temporal stages of regeneration [9].

Currently, oral corticosteroids, such as prednisone and deflazacort, are the standard of care for DMD [10]. They improve muscle strength, functional outcomes as well as longer survival. However, there are significant adverse effects for long term due to the general deactivation of the immune system. Those include excessive weight gain, high blood pressure, adrenal insufficiency, stunted growth, cushingoid appearance, behavioral changes, decreased bone mineral density and increased incidence of fractures [11].

Although novel dissociative steroids may be superior substitutes to corticosteroids because they showed significantly reduced adverse effects and avoid growth stunting [12], other potential therapeutics should be explored. Repurposing or developing novel pharmacological therapies capable of addressing the many pathogenic features of DMD in addition to anti-inflammation could elicit greater therapeutic advantages. We hypothesize that targeting directly the molecule triggering the muscle inflammatory response can help reduce the current therapy side effects while keeping the benefits of the reduced inflammation.

Cytokines play a major role in mediating muscular inflammation. High levels of pro-inflammatory cytokines such as tumor necrosis factor-alpha (TNF-α), interleukin 1 beta (IL-1β), interleukin 6 (IL-6), among others, are present in DMD patients tissues and serum samples [13–14]. IL-1β belongs to the IL-1 family, and macrophages produce it. The protein is a mediator of the inflammatory response and is involved in cell proliferation, differentiation, and apoptosis. On the other hand, IL-6 is an interleukin that can act as a pro-inflammatory and anti-inflammatory molecule, depending on its upstream and downstream signaling. It is considered a myokine, a cytokine produced in the muscle and is elevated due to muscular contraction. It is believed that IL-6 inflammatory response can help both in muscle repair and contribute to muscular damage [15–16]. Our group as well as others have shown that both the IL-1β and IL-6 pathways are interconnected. Treatment of myoblasts with IL-1β results in the release of pro-inflammatory IL-6 from myoblasts in a time and dose-dependent manner [17–18].

We have also previously demonstrated that normal primary skeletal muscle cells are capable of secreting IL-1β in response to combined treatment with lipopolysaccharide (LPS) and the P2X7 receptor agonist, benzylated-ATP. Our data suggest that muscle cells in addition to immune cells can play an active role in promoting inflammatory processes in genetic muscle diseases such as DMD and LGMD2B [19]. Our group also has shown that IL-1β is up-regulated in dystrophin-deficient muscle in mdx mice and treatment with IL-1 receptor antagonist (IL-1Ra) Kineret®, resulted in partial inhibition of IL-1β with no functional benefits in these mice [20]. Our group and others have also pursued direct inhibition of IL-6 signaling pathways in mdx mice with conflicting outcomes [4,21].

Canakinumab or ILARIS®, a human monoclonal antibody targeted specifically at IL-1β, is approved for the treatment of autoinflammatory conditions such as Periodic Fever Syndromes (e.g., Cryopyrin-Associated Periodic Syndromes, Tumor Necrosis Factor Receptor Associated Periodic Syndrome, Familial Mediterranean Fever) and Systemic Juvenile Idiopathic Arthritis [22–23]. Also, some studies have shown significantly lower recurrent cardiovascular events after Canakinumab treatment [24]. Canakinumab prevents the binding of endogenous human IL-1β to its cognate receptor on its target cells surface, thus functionally neutralizing its pro-inflammatory bioactivity. It does not bind to human IL-lα or the endogenous antagonist IL-1Ra [22].

We hypothesized that Canakinumab may be more effective in blocking inflammatory cascade in dystrophin-deficient muscle because a) Some studies in Muckle-Wells syndrome patients have shown that treatment with canakinumab was superior to anakinra, which directly competes for binding to the IL-1β receptor, therefore blocking the biological activity of IL-1 [5], b) Canakinumab does not compromise the biological activity of IL-1Ra, an important endogenous inhibitor of IL-1 activity in vivo and the inhibitory effect of canakinumab and IL-1Ra likely to be additive c) Canakinumab is highly specific for IL-1β, and does not interfere with other IL-1-activated pathways in contrast anakinra, blocks the signals of both the IL-1α and IL-1β isoforms and d) Canakinumab due to its longer half-life, can block IL-1β action for a longer time without the need for frequent injections or high doses [6].

In this study, we investigated canakinumab as a potential inhibitor of pro-inflammatory cytokine IL-6 in human skeletal muscle cells. Our results demonstrate that canakinumab is a potent inhibitor of IL-6 production in IL-1β-stimulated human myoblasts and myotubes. These results support further investigation into the clinical utility of repositioning canakinumab for the treatment of DMD.

## Materials and methods

### Cell culture

Primary human Skeletal Muscle Cells (SkMC) (C-12530, Lot# 427Z005, Promocell; PCS-950-010 Lot# 80326194, ATCC) and maintained with Ham’s F-10 Nutrient Mix, HEPES (ThermoFisher Scientific) supplemented with 20% fetal bovine serum (FBS) (ThermoFisher Scientific), 2% Chick Embryo Extract (CEE) (USBiological Life Sciences), and 1% Penicillin-Streptomycin (P/S) (ThermoFisher Scientific) at 37°C. SkMCs myoblasts were maintained in T175 flasks coated with 0.4% sterile Gelatin solution (Sigma-Aldrich) or Matrigel (Corning) at a plating density of 3000 to 7000 cells/cm^2^ and replenished with fresh medium every 2-3 days after the initial round of expansion. For the experiments, SkMCs myoblasts were plated in 96-well Tissue culture (TC)-treated microplates at a density of 10,000 cells per well (100 µl) and allowed to grow for 24 hrs before further experimentation.

Human leukemic monocyte cell line (THP-1) was received from the laboratory of Dr. Kanneboyina Nagaraju (Children’s National Medical Center, Washington D.C.) and maintained in RPMI-1600 media (ATCC) supplemented with 10% FBS, 1% P/S, and 0.05 mM β-Mercaptoethanol (β-ME) (Sigma-Aldrich). THP-1 cells were maintained at 37°C in T75 flasks at a plating density of 4 - 8x105 cells/mL and replenished with fresh medium every 2-3 days after the initial round expansion. These cells were replenished with medium every 2-3 days and passaged bi-weekly. To induce differentiation, the growth media was removed, and the cells were washed twice with DPBS (Thermofisher, 14190144), prior to adding the insulin-rich differentiation media (DMEM with 2% of Horse Serum and Gentamicin 50 µg/ml). The cells were allowed to differentiate for 10-12 days with regular media changes every 3-4 days. THP-1 cells were plated in 96-well TC-treated microplates at a density of 10,000 cells per well (100 µl) and allowed to grow for 24 hrs before further experimentation.

### Immunohistochemistry and supernatant/media collection

Myogenecity of the SkMC myoblasts was assessed by direct immunofluorescent staining for the myoblast marker desmin, using a monoclonal anti-desmin antibody produced in mice. All reagents were obtained from Sigma-Aldrich Co (St Louis, MO, USA), otherwise indicated. SkMC myoblasts were plated in 8-chamber slides and allowed to adhere for 24 hrs. Before cell fixation, we collected the supernatant for further usage. It was centrifuged at 1,000 g for 15 min at 4°C to eliminate debris in the pellet and aliquoted in 100 µl for further use.

**Fig 1:**
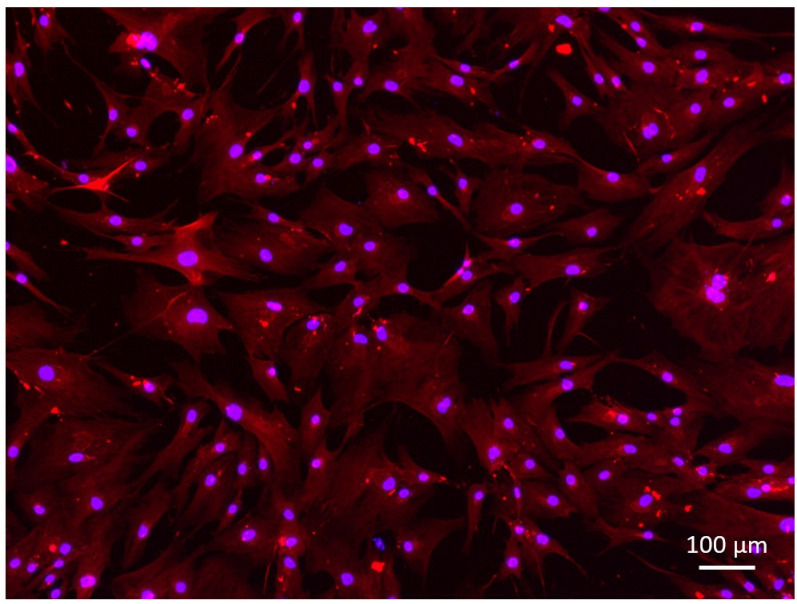
Desmin staining of primary human myoblasts (20x magnification). Most of the cells stained positive for desmin, suggesting that these are myogenic cells. Bar =  100 µm.

Cultures were fixed with 4% cold paraformaldehyde in PBS for 10 mins, then washed in phosphate buffer saline (PBS) containing 1% Triton X. Cultures were then blocked with PBS containing 10% sheep serum and 1% Triton-X for 75 mins at room temperature. For desmin, cultures were incubated overnight at 4°C with monoclonal mouse anti-desmin Immunoglobulin (Ig) G-1 antibody (clone DE-U-10, D1033) diluted 1:100 in PBS with 2% sheep serum and 1% Triton-X. After 3 washes with 1% Triton-X in PBS for 10 mins each, cultures were incubated with a secondary antibody, goat anti-mouse IgG12 Alexa 488 (A-21121, ThermoFisher Scientific), diluted 1:200 in PBS with 2% sheep serum, and 1% Triton-X for 1 hr at room temperature in the dark. Cultures were washed three times with 1% Triton-X in PBS for 10 mins each. Slides were mounted in histology mounting medium, Fluoroshield^TM^ with DAPI (F6057, Millipore-Sigma) to stain nuclei and stored at 4°C before analysis (**Fig 1**).

### Treatment preparation

IL-1β was reconstituted at 25 µg/mL in a sterile PBS +  0.1% BSA (Pierce) solution and stored at -20°C. A stock solution of lipopolysaccharides (LPS) from Escherichia coli 0111:B4 (Sigma-Aldrich) was aliquoted at 1 mg/ml and stored at -20°C. Canakinumab was obtained as a solution at 150 mg/ml (1.03 mM) from Novartis and then a biosimilar was purchased (MAB10349-100, R&D Systems). Recombinant Human IL-1β protein was purchased from R&D Biosystems as a lyophilized 5µg powder.

To prepare the treatment following the co-incubation scheme, the working solutions of canakinumab and IL-1β were mixed in a tube and placed horizontally in an orbital shaker at 300 rpm for 30 minutes. Then, media was added to obtain the final concentration and was dispensed on the 96-well plates (100 µl) or the chamber slides (300 µl). The media control and the single treatments with canakinumab and IL-1β were also added at this time. For the pretreatment scheme, the SkMCs cells were treated with canakinumab for 30 min before administering the IL-1β, and the rest of the media/treatments in the rest of the wells.

### Cell proliferation and viability assay

Cell Proliferation Kit I (MTT Assay, Sigma-Aldrich) and CellTiter 96® Non-Radioactive Cell Proliferation Assay (MTT, Promega) were used for the non-radioactive quantification of cell proliferation and viability. Myoblasts and myotubes were grown in microplates in a volume of 100 µl culture medium/well with a plating density of 5-7,000 cells/well to perform the assay. These cells were given 24 hrs to acclimatize before the start of assay either as adherence (SkMCs myoblasts) or floating cells (THP-1). The assays were conducted as per the manufacturer’s instructions. The samples’ spectrophotometrical absorbance was measured using an Epoch 2 microplate spectrophotometer (spectrum scanning between 550-600 nm with a reference wavelength of 650 nm). Raw data was saved as a.xpt file on the Gen5 software and exported as an excel file for further analysis.

### Human IL-6 Enzyme-Linked Immunosorbent Assay (ELISA)

Human IL-6 DuoSet ELISA kit (DY206) and DuoSet Ancillary Reagent Kit 2 (DY008) were purchased from R&D Biosystems to develop sandwich ELISAs to measure human IL-6. SkMCs myoblasts, myotubes and THP-1 cells were grown in microplates in a volume of 100 µl culture medium/well with a plating density of 10,000 cells/well to perform the assay. These cells were given 24 hrs to attach to the plate matrix before the start of the assay. These cells were treated at their respective time points. Upon incubation completion the cell media was collected and stored at -80°C. According to the manufacturer’s instructions, ELISA was performed on the samples following the appropriate dilution (1:500). It was read using an Epoch 2 microplate spectrophotometer (at 450 nm with two-wavelength corrections at 540 nm). Raw data was saved as a.xpt file on the Gen5 (Version 3.11) software and exported as an excel file and GraphPad Prism v9 for further analysis and graph plotting. IL-6 production was normalized to the number of cells initially seeded.

### Statistical analysis

The nominal level for defining statistical significance is p ≤  0.05. Normality is tested for each treatment group’s outcome using both the Shapiro-Wilk normality test and a visual inspection of each histogram. Analysis of Inhibition dose-response will use a non-linear regression model to calculate IC_50_, per treatment time point.

## Results

### *Recombinant IL-1*β *has no toxic effects on SkMCs myoblasts*

**Fig 2:**
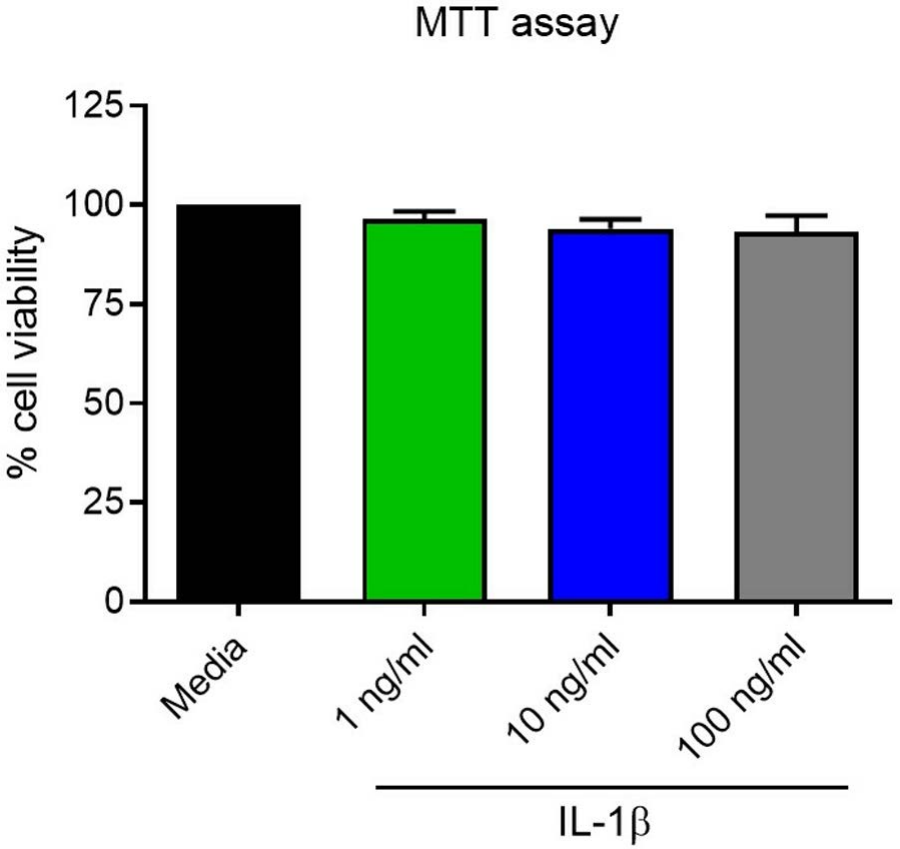
Cell viability (MTT) assay for four conditions - media control and three concentrations of IL-1 β (1 ng/ml, 10 ng/ml, 100 ng/ml) after 72 hrs of treatment (n = 3 biological replicates, n = 8 technical replicates, media vs treatment groups is not significant, p = ns). Data shown as mean ±  SEM.

We treated SkMCs myoblasts with different concentrations of IL-1β to assess their cytotoxicity. Three biological replicates from SkMCs myoblasts were prepared for four different treatment conditions: an untreated “media-control” group and 3 IL-1β groups: 1 ng/ml, 10 ng/ml, and 100 ng/ml (n > 4 technical replicates). At 72 hrs, an MTT assay was performed to assess the viability of the cells’ exposure to IL-1β. We found that the viability of SkMCs myoblasts was not significantly affected (p = ns) compared to the control group, even at the higher dose of 100 ng/ml of IL-1β (**Fig 2** and **Tables S1-2**).

### IL-6 production *in* response *to* IL-1β treatment *in* myoblasts

Unstimulated SkMCs myoblasts produce minimal IL-6, but upon stimulation with either of IL-1β or LPS the IL-6 production significantly increases. To set up a positive control for IL-6 production, we treated THP-1 monocytic cells with LPS. LPS triggers an immunological response from the cells by promoting IL-6 production [25]. An ELISA was used to detect and quantify the amount of inflammatory cytokine IL-6 produced in the supernatant of SkMCs myoblasts and THP-1 cells. The THP-1 control showed a 15-fold increase in IL-6 production when stimulated for 48 hrs by 1 µg/ml of LPS (p = 0.0023). Before measuring the IL-6 concentrations of the rest of the treatments, the controls were serially diluted (1/10, 1/20, 1/40, 1/80, 1/160, 1/320, 1/640) to determine optimal dilution for the ELISA. Since the best dilutions for detecting IL-6 were 1/320 and 1/640, we decided to move forward with a 1/500 dilution of the cell supernatant when measuring IL-6 by ELISA (**Figure S1** and **Tables S3-5**).

We then performed a time- and dose-response experiment, stimulating SkMCs myoblasts with different doses of IL-1β, ranging from 0 (media) to 100 ng/mL and times ranging from 6 to 72 hours. The SkMCs myoblasts were tested with four treatment conditions: an untreated “media-control” group, an IL-1β-1 ng/ml group, an IL-1β-10 ng/ml group, and an IL-1β-100 ng/ml group (n > 4 technical replicates, n = 3 biological replicates). These four supernatants were collected at four different time points (6 hrs, 24 hrs, 48 hrs, and 72 hrs). Positive control of the human leukemic monocyte cell line (THP-1) was included with two treatment conditions: a media control group and an LPS (1 µg/ml) treated group (n > 4 technical replicates) for 48 hrs.

**Fig 3:**
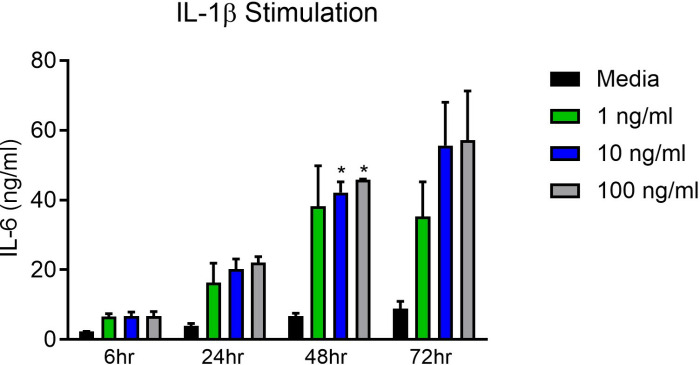
Treatment with IL-1 β triggers IL-6 production in SkMC myoblasts. Treatment of SkMCs myoblasts with IL-1β increases IL-6 compared to media alone. We observed a significant increase of IL-6 after 48h and with both 10 and 100 ng/ml of IL-1β (p < 0.05) (n = 2 biological replicates, n = 8 technical replicates). Individual graphs for each timepoint and dose are provided in the supplementary data (**Fig** **S2** and **Tables S15-22**; and **Fig** **S3** and **Tables S23-30**, respectively).

Treatment of myoblasts with 10 and 100 ng/ml of IL-1β for 48 hrs significantly increased IL-6 production compared to media alone (p < 0.05) (**Fig 3** and **Tables S6-14**). Given that the level of IL-6 production was similar between 10 and 100 ng/ml of IL-1β, 10 ng/ml of IL-1β dosage was used to assess the efficacy of canakinumab in further experiments. We also determined that the optimal time is between 48 and 72 hours for further experiments.

We observed both an IL-1β and time dependent increase in the production of IL-6.

### Viability *of* myoblasts *after* canakinumab treatment

**Fig 4:**
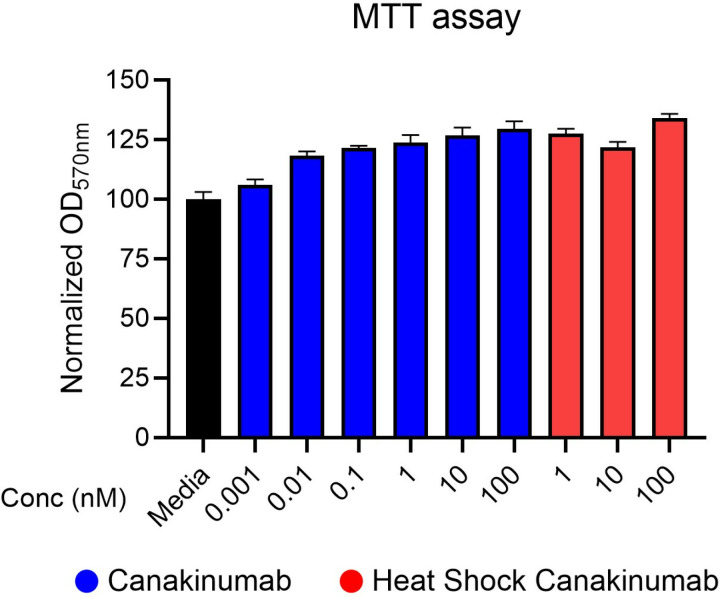
Cell viability analysis after canakinumab treatment for 72 hr. MTT assay for ten conditions- media control, six concentrations of canakinumab (0.001 nm, 0.01 nm, 0.1 nm, 1 nm, 10 nm, 100 nm) and three concentrations of 1 hr heat-shocked canakinumab (1 nm, 10 nm, and 100 nm) after 72 hrs of treatment (n = 2 biological replicates, n = 8 technical replicates, p = ns).

To determine the possible cell toxicity of canakinumab, two biological replicates from SkMCs myoblasts were prepared for ten 72 hrs treatment conditions: an untreated “media-control” group, 0.001 nM canakinumab, 0.01 nM canakinumab, 0.1 nM canakinumab, 1 nM canakinumab, 10 nM canakinumab, 100 nM canakinumab, 1 nM Heat-Inactivated canakinumab, 10 nM Heat-Inactivated canakinumab, and 100 nM Heat-Inactivated canakinumab (n > 4 technical replicates). An MTT assay was performed to evaluate cell viability. No significant cell viability changes were observed using any of the tested treatment conditions (**Fig 4** and **Tables S31-32**). The results indicated that the drug is well tolerated by myoblasts at the 6 concentrations tested.

### Canakinumab *inhibits IL-6 production in human myoblasts*

Two biological replicates from SkMCs myoblasts were prepared for 12 treatment conditions following two treatment schemes for 48 hrs with either media control or IL-1β (10 ng/ml): treating SkMCs myoblasts with canakinumab 30 mins before administering IL-1β or incubating canakinumab with IL-1β 30 mins before administration. We hypothesize that co-incubation of IL-1β with canakinumab would have a greater effect since the cytokine is blocked before it can even reach its receptor.

**Fig 5:**
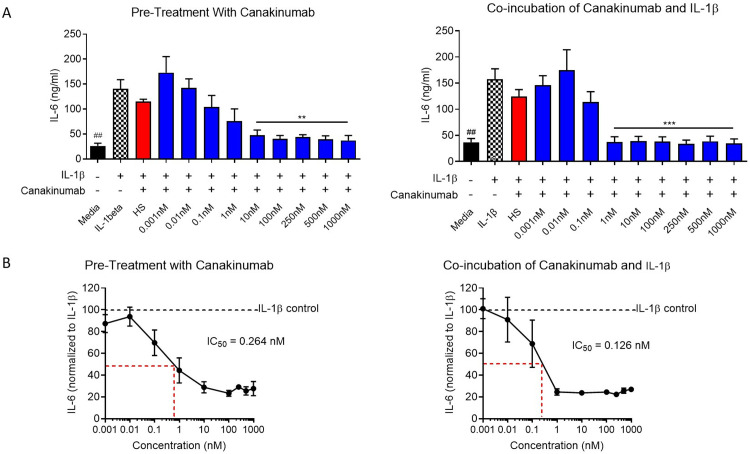
IL-6 ELISA measurements for 12 treatment conditions (media controls, canakinumab (0.001 nm, 0.01 nM, 0.1 nM, 1 nM, 10 nM, 100 nM, 250 nM, 500 nM, and 1000 nM), and 1-hr 100 nM heat-shocked canakinumab stimulated with IL-1 β using two treatment schedules for 48 h. The left graphs show 30 min pre-treatment with canakinumab before administering 10 ng/ml of IL-1β for 48 hrs. The right graphs indicate treatment with a 30 min co-incubation of canakinumab with 10 ng/ml of IL-1β before administration of solution to myoblasts for 48 hrs. **A)** Measured IL-6 (ng/ml) per treatment condition. **B)** IC_50_ calculation using 8 different concentrations of canakinumab treatment. For all assays shown above, n = 2 biological replicates, n = 4 technical replicates.

Canakinumab concentrations were 0.001 nM, 0.01 nM, 0.1 nM, 1 nM, 10 nM, 100 nM, 250 nM, 500 nM, 1000 nM and heat-inactivated 100 nM. Additionally, a media control was included. Human IL-6 was measured in the supernatant of the treated myoblasts. IL-6 levels in the IL-1β control were significantly higher than those in the media control, both in the pre-treatment scheme (p = 0.0003) and the co-incubation scheme (p < 0.0001). No significant differences were observed between IL-1β control and 1 hr heat-shocked canakinumab at 100 nM. Results indicated that the total IL-6 detected in each biological replicate was significantly reduced relative to IL-1β stimulated control at 1 nM, 10 nM, 100 nM, 250 nM, 500 nM, and 1000 nM using both treatments schemes (**p < 0.01, ***p < 0.001; respectively) (**Fig 5A** and **Tables S33-36**). The IC_50_ of pre-treatment with canakinumab was calculated to be over 2-folds greater (0.264 nM) than co-incubation of canakinumab with IL-1β (0.126 nM), suggesting a greater effect using the co-incubation scheme (**Fig 5B** and **Tables S37-38**). Taken together, we have shown that canakinumab is a potent inhibitor of IL-6 production in IL-1β-stimulated human myoblasts.

### *Effects of IL-1β,* canakinumab and combination treatment in *SkMCs myotubes*

**Fig 6:**
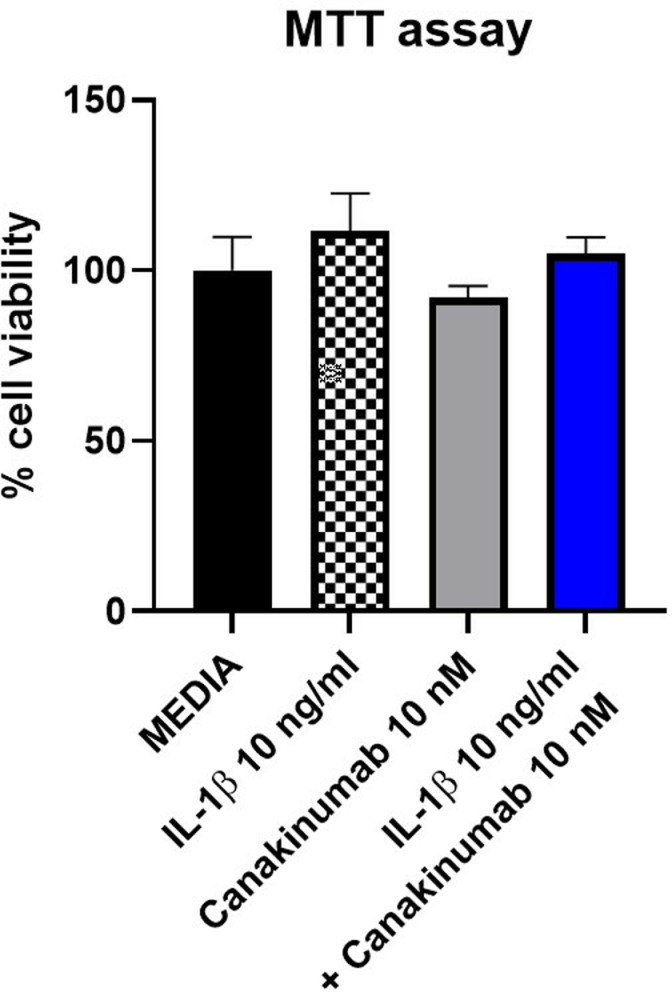
Cell viability (MTT) assay for four conditions - media control, IL-1 β 10 ng/ml, Canakinumab 10 nM, and the co-incubation of IL-1β 10 ng/ml and canakinumab 10 nM after 72 hrs of treatment (n = 2 biological replicates, n = 3 technical replicates). Data shown as mean ±  SEM.

After observing promising data in the assays in myoblasts, we wanted to investigate the effects of canakinumab in myotubes, the functional contractile cells in the muscle tissue. To assess toxicity of the three treatments, the differentiated myotubes were treated under four conditions: “media-control” group, IL-1β 10 ng/ml, canakinumab 10 nM and the co-incubation of IL-1β 10 ng/ml and canakinumab 10 nM for 72 hours (biological replicates n = 2, technical replicates n = 3). The concentrations were selected based on the experiments in myoblasts. No significative differences were observed within the three groups (p = ns) (**Fig 6** and **Tables S39-40**).

### Canakinumab inhibits IL-6 production *in* human myotubes

Two biological replicates from SkMCs myoblasts were prepared for four treatment conditions and treated for 48 hrs with either media control, or IL-1β (10 ng/ml), canakinumab 10 nM or IL-1β 10 ng/ml and canakinumab 10 nM following the co-incubation scheme.

**Fig 7:**
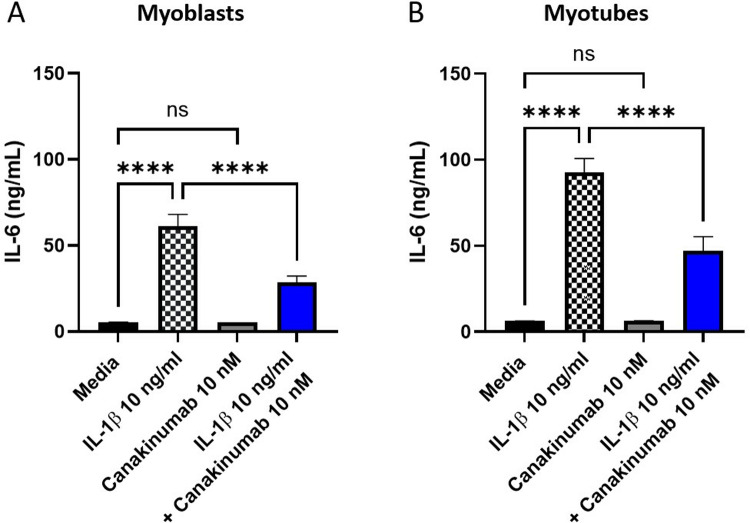
Evaluation of the effects of canakinumab in reducing IL-6 production in myoblasts (A) and myotubes, the terminally differentiated and functional muscle cells **(B)**. The ELISA assay showed significant reduced levels of IL-6 after 30 min IL-1β (10 ng/ml) coincubation with canakinumab (10 nM) in both cell types (p < 0.0001).

Human IL-6 was measured in the supernatant of the treated myotubes in the same fashion we did for the myoblasts. IL-6 levels in the myoblasts and myotubes treated with IL-1β were significantly higher than those in the media control (p < 0.0001). No significant differences were observed between the media control and 10 nM canakinumab. A significant reduction of IL-6 was observed in the cells treated with IL-1β 10 ng/ml and canakinumab 10 nM following the co-incubation scheme in comparison to IL-1β stimulated control (p < 0.0001) (**Fig 7** and **Tables S41-44**). This data suggests that canakinumab is a potent neutralizer of IL-1β signaling in human myoblasts and myotubes.

## Discussion

In chronic inflammatory diseases such as DMD, cells’ responses to the inflammatory stimuli, which are characterized collectively as inflammasome, result in the production of pro-inflammatory cytokines such as IL-1β [18]. The inflammasome is a multimeric protein complex that leads to the activation of inflammatory responses and has been well characterized in cells to participate in innate immunity [19]. Both myoblasts and inflammatory cells are believed to orchestrate inflammatory responses in the skeletal muscle [26]. In this study, we assessed the use of canakinumab as a potential anti-inflammatory agent to inhibit IL-1β production and reduce IL-6 levels.

First, we initiated the proof of concept that the presence of IL-1β resulted in the release of IL-6 in SkMCs myoblasts and that treating the SkMCs myoblasts with IL-1β did not lead to toxicity of the cells. The data showed that subjecting SkMCs myoblasts to IL-1β resulted in a dose-dependent increase in the IL-6 production, one of IL-1β downstream metabolites, thereby mimicking the inflammatory signaling. This demonstrated the connection between IL-1β and IL-6 expression and reassured the rationale for the canakinumab treatment. Subsequently and after showing that canakinumab was not toxic to cells under concentration below or equal to 100 nM, the SkMc myoblasts were treated with IL-1β combined with its inhibitor, canakinumab. This showed a reduction in the amounts of IL-6 present in the media with inhibitor concentrations equal to or above 1 nM. We also carried out two treatment schemes using canakinumab one featuring a co-incubation of canakinumab and IL-1β and a pre-treatment of canakinumab In comparison, both methods proved to be effective in reducing the IL-6 levels in the supernatant (p < 0.0001), the half-maximal inhibitory concentration (IC_50_) of the co-incubation of canakinumab with IL-1β was lower (0.126 nM), suggesting higher inhibitory efficiency when canakinumab is first bound to the IL-1β. We also performed toxicity assays in myotubes, the functional mature cell type in the human body showing no signs affecting cell viability. We also assessed the IL-6 production as a response to IL-1β stimulation and canakinumab treatment. Myotubes treated with canakinumab didn’t show significant differences in IL-6 production in comparison with the control myotubes. Myotubes treated with IL-1β showed a significant increase in IL-6 production (p < 0.0001), but when the myotubes were treated with IL-1β co-incubated with canakinumab, Il-6 production was significantly reduced in comparison to those treated with IL-1β alone (p < 0.001). To our knowledge, this is the first study to show that canakinumab can neutralize the production of IL-1β, and as a result, reduce the IL-6 levels in cells that are not inflammatory.

The IC_50_ is the measure of the potency of a compound in inhibiting a specific biological process. In a cell-based assay system with human primary fibroblasts, the IC50 for neutralization of human recombinant IL-1β using canakinumab was 43.6 ±  5.5 pM [27]. In vivo, it is known that canakinumab binds to circulating IL-1β forming a complex. Since the complex renders a large molecular size complex, it is foreseen to take longer to be eliminated in comparison to the free IL-1β, causing increased total IL-1β levels [28]. Our research using SkMCs myoblasts obtained an IC_50_of 264 pM when there was pre-treatment, and an IC_50_of 126 pM when canakinumab was co-incubated with the IL-1β, implying a higher effect using the co-incubation scheme. These results align with the previous data, with a lower IC_50_ when canakinumab is co-incubated with IL-1β. The values may differ due to the nature of the cells used, primary fibroblasts versus immortalized myoblasts, but remain in a similar order of magnitude.

Our study’s limitation is the inability to perform in vivo studies in the mdx DMD mouse model. Canakinumab does not cross-react with IL-1β from mouse, rat, rabbit, rhesus, or cynomolgus monkey. The only non-human species specifically recognized by canakinumab is the marmoset monkey. This limited species cross-reactivity can be explained with one amino acid in the IL-1β sequence crucial for canakinumab binding. At position 64, Glu is present in human and marmoset IL-1β but changed to Ala or Gly in all other species analyzed. This amino acid has a crucial role in antibody-antigen recognition while outside the binding domain [27].

Although there is a consensus that the IL-1 family is pro-inflammatory, available data supports the dual role of IL-6 as a pro-inflammatory cytokine and an anti-inflammatory myokine. A high level of IL-6 has been shown to have detrimental effects (pro-inflammatory) depending on the amount and the length of time the cytokine exists. One study has shown that low IL-6 doses would be stimulatory/homeostatic in a short period, whereas high prolonged doses would be pathologic [25]. This potentially explains the beneficial IL-6 expression during muscular exercise and the results in muscle damage due to the repeated and prolonged accumulation of IL-6 observed in DMD. Consistently, a study in a muscle atrophy by tail suspension mouse model has shown that inhibition of IL-6 production significantly reduced detrimental effects on skeletal muscle [29].

Up to date, canakinumab has been successfully used in several inflammatory-related diseases such as systemic juvenile idiopathic arthritis [30], Adult-Onset Still’s Disease [31], atherosclerosis [32], pyoderma gangrenosum [33], and Familial Mediterranean fever [18], among others, which all have in common aberrant IL-1 signaling. While the development of new drugs is a lengthy and expensive process, repurposing already existing drugs can reduce the time and the cost of the treatments. canakinumab is already approved and available, with known safety, toxicity profiles, and dosage information. This should help to accelerate the testing of such a drug in patients [34]. Given the potential beneficial effects of blocking IL-1β and reducing the circulating IL-6 level in chronic inflammatory diseases, this drug can be repurposed for muscular conditions such as DMD.

## Supporting information

S1 Fig IL-6 ELISA optimization. (A) IL-6 ELISA for Human leukemic monocyte cell line (THP-1) controls. (B) ELISA curve for IL-6 standards: 0, 9.38, 48.8, 37.5, 75, 150, 300 and 600 pg/ml. (C) IL-6 ELISA optimization via serial dilutions for SkMC supernatant treated with IL-1β-100 ng/ml for 24h. (TIP)

S2 Fig Cell viability (MTT) assay for four timepoints. (A) 6 h, (B) 24 h, (C) 48 h and (D) 72 h. In each timepoint media control and three concentrations of IL-1β (1, 10 and 100 ng/ml) were tested for 72 hrs of treatment (n = 3 biological replicates, n = 8 technical replicate We observed a significant increase of IL-6 after 48h and with both 10 and 100 ng/ml of IL-1β (p < 0.05). Data shown as mean ±  SEM. (TIP)

S3 Fig Cell viability (MTT) assay for four treatment doses. (A) Control media, (B) 1 ng/ml, (C) 10 ng/ml and (D) 100 ng/ml. For each dose, four timepoints were tested (6h, 24h, 48h, 72h) for 72 hrs of treatment (n = 3 biological replicates, n = 8 technical replicates, media vs treatment groups are not significant, p = ns). Data shown as mean ±  SEM. (TIF)

File S1 Supporting data: Table S1. Data for Figure 2 MTT assay. Table S2. Data analysis results for Figure 2. Ordinary one-way ANOVA with post-hoc Tukey test. Table S3. Data for Figure S1A THP-1 monocytes control. Table S4. Data for Figure S1B Standard curve example. Table S5. Data for Figure S1C Sample dilution optimization. Table S6. Data for Figure 3 IL-1β stimulation. Table S7. Data analysis results for Figure 3 at 6h of treatment. Ordinary one-way ANOVA with post-hoc Tukey test. Table S8. Data analysis results for Figure 3 at 24h of treatment. Ordinary one-way ANOVA with post-hoc Tukey test. Table S9. Data analysis results for Figure 3 at 48h of treatment. Ordinary one-way ANOVA with post-hoc Tukey test. Table S10. Data analysis results for Figure 3 at 72h of treatment. Ordinary one-way ANOVA with post-hoc Tukey test. Table S11. Data analysis results for Figure 3 for media treatment. Kluskal-Wallis test with post-hoc Dunn’s test. Table S12. Data analysis results for Figure 3 for 1 ng/ml IL-1β treatment. Kluskal-Wallis test with post-hoc Dunn’s test. Table S13. Data analysis results for Figure 3 for 10 ng/ml IL-1β treatment. Kluskal-Wallis test with post-hoc Dunn’s test. Table S14. Data analysis results for Figure 3 for 100 ng/ml IL-1β treatment. Kluskal-Wallis test with post-hoc Dunn’s test. Table S15. Data for Figure S2A at 6h of treatment. Table S16. Data analysis results for Figure S2A at 6h of treatment. Ordinary one-way ANOVA with post-hoc Tukey test. Table S17. Data for Figure S2B at 24h of treatment. Table S18. Data analysis results for Figure S2B at 24h of treatment. Ordinary one-way ANOVA with post-hoc Tukey test. Table S19. Data for Figure S2C at 48h of treatment. Table S20. Data analysis results for Figure S2C at 48h of treatment. Ordinary one-way ANOVA with post-hoc Tukey test. Table S21. Data for Figure S2D at 72h of treatment. Table S22. Data analysis results for Figure S2D at 72h of treatment. Ordinary one-way ANOVA with post-hoc Tukey test. Table S23. Data for Figure S3A for media treatment. Table S24. Data analysis results for Figure S3A for media treatment. Kluskal-Wallis test with post-hoc Dunn’s test. Table S25. Data for Figure S3B for 1 ng/ml IL-1β treatment. Table S26. Data analysis results for Figure S3B for 1 ng/ml IL-1β treatment. Kluskal-Wallis test with post-hoc Dunn’s test. Table S27. Data for Figure S3C for 10 ng/ml IL-1β treatment. Table S28. Data analysis results for Figure S3C for 10 ng/ml IL-1β treatment. Kluskal-Wallis test with post-hoc Dunn’s test. Table S29. Data for Figure S3D for 100 ng/ml IL-1β treatment. Table S30. Data analysis results for Figure S3D for 100 ng/ml IL-1β treatment. Kluskal-Wallis test with post-hoc Dunn’s test. Table S31. Data for Figure 4 MTT assay. Table S32. Data analysis results for Figure 4. Kluskal-Wallis test with post-hoc Dunn’s test. Table S33. Data for Figure 5A for pre-treatment with canakinumab. Table S34. Data analysis results for Figure 5A for pre-treatment with canakinumab. Ordinary one-way ANOVA with post-hoc Tukey test. Table S35. Data for Figure 5A for co-incubation of canakinumab and IL-1β. Table S36. Data analysis results for Figure 5A for co-incubation of canakinumab and IL-1β. Ordinary one-way ANOVA with post-hoc Tukey test. Table S37. Data used for Figure 5B for pre-treatment with canakinumab. Analysis of Inhibition dose-response using a non-linear regression model to calculate IC50, per treatment time point. Table S38. Data used for Figure 5B for co-incubation of canakinumab and IL-1β. Analysis of Inhibition dose-response using a non-linear regression model to calculate IC50, per treatment time point. Table S39. Data for Figure 6 MTT assay. Table S40. Data analysis results for Figure 6. Ordinary one-way ANOVA with post-hoc Dunnett’s multiple comparisons test. Table S41. Data for Figure 7A myoblasts. Table S42. Data analysis results for Figure 7A. Ordinary one-way ANOVA with post-hoc Tukey test. Table S43. Data for Figure 7B myotubes. Table S44. Data analysis results for Figure 7B. Ordinary one-way ANOVA with post-hoc Tukey test.
(PDF)
