# Supplementary figures and images for: The effect of IL-1β inhibitor canakinumab (Ilaris®) on IL-6 production in human skeletal muscle cells
Source: PLoS One. 2025 Mar 6;20(3):e0316110. doi: 10.1371/journal.pone.0316110 (PMC11884680; doi:10.1371/journal.pone.0316110)

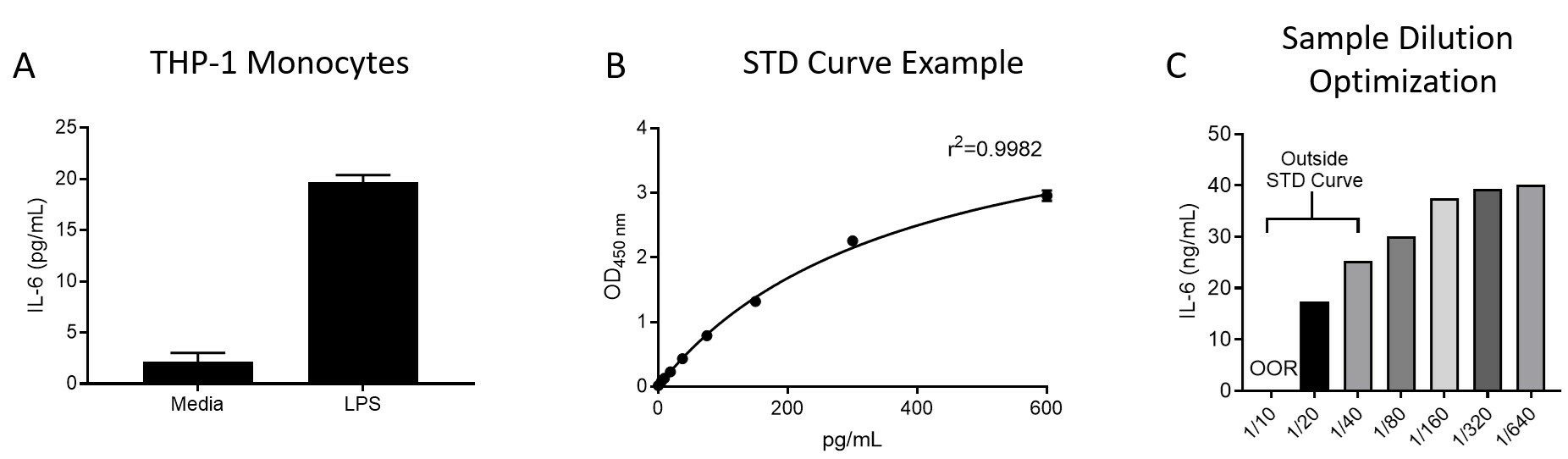

Supplement: S1 Fig — (TIP) [file pone.0316110.s001.tif]

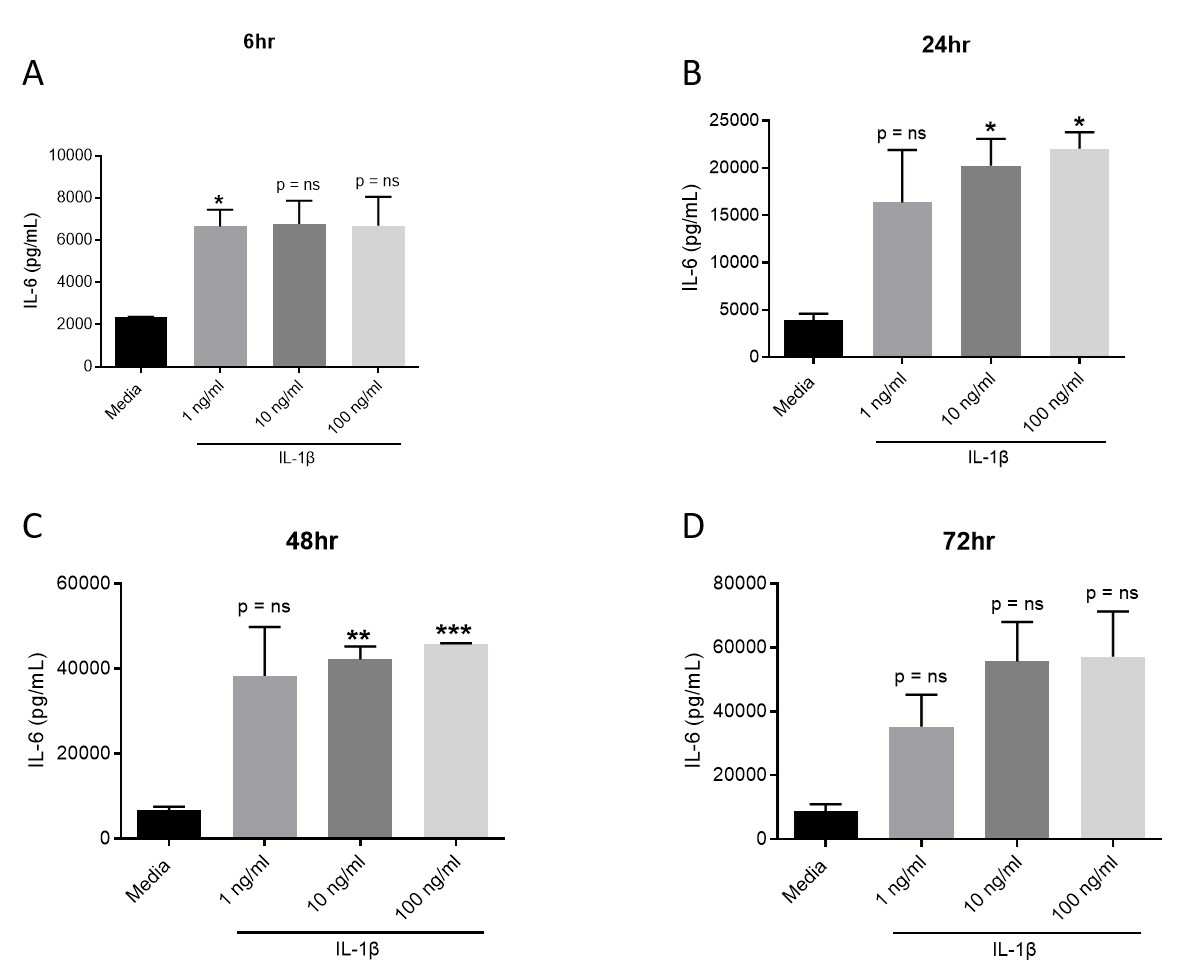

Supplement: S2 Fig — (TIP) [file pone.0316110.s002.tif]

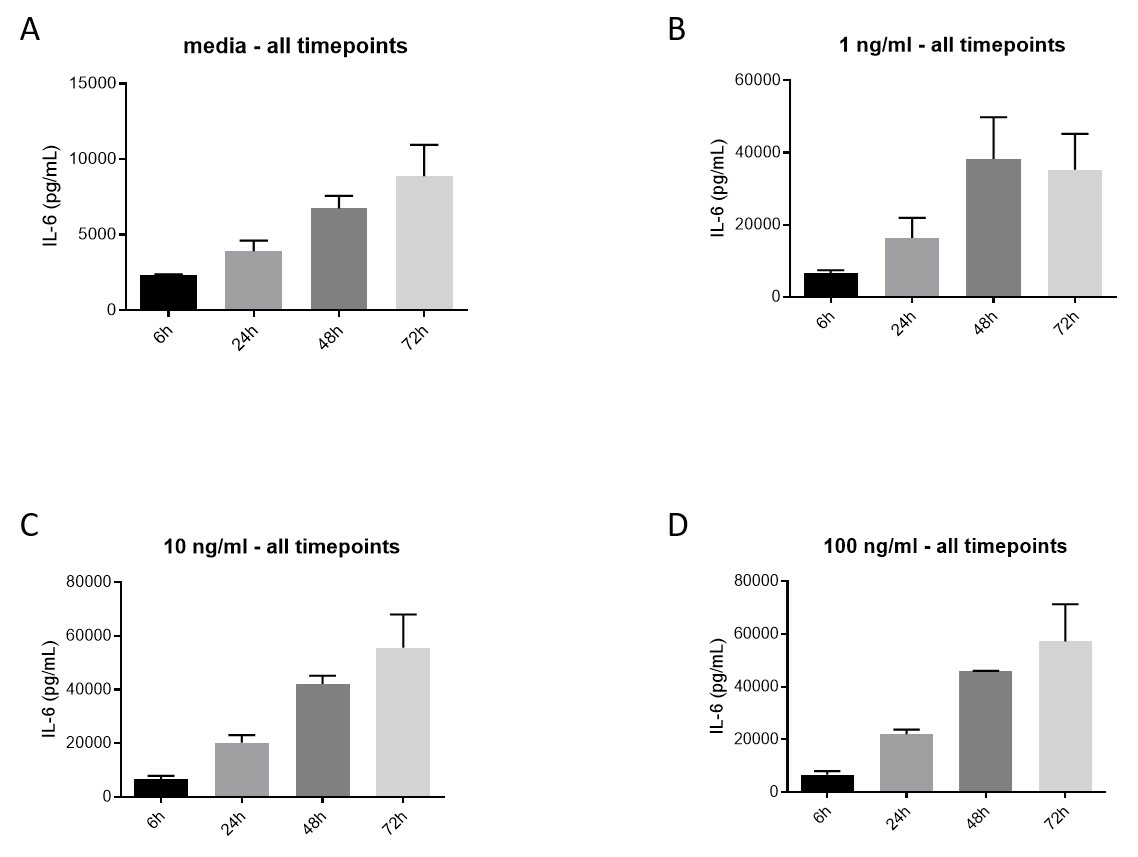

Supplement: S3 Fig — (TIF) [file pone.0316110.s003.tif]
