# Supplementary material for: The effect of IL-1β inhibitor canakinumab (Ilaris®) on IL-6 production in human skeletal muscle cells
Source: PLoS One. 2025 Mar 6;20(3):e0316110. doi: 10.1371/journal.pone.0316110 (PMC11884680; doi:10.1371/journal.pone.0316110)
Supplement: File S1 — Supporting data: Table S1. Data for Figure 2 MTT assay. Table S2. Data analysis results for Figure 2. Ordinary one-way ANOVA with post-hoc Tukey test. Table S3. Data for Figure S1A THP-1 monocytes control. Table S4. Data for Figure S1B Standard curve example. Table S5. Data for Figure S1C Sample dilution optimization. Table S6. Data for Figure 3 IL-1β stimulation. Table S7. Data analysis results for Figure 3 at 6h of treatment. Ordinary one-way ANOVA with post-hoc Tukey test. Table S8. Data analysis results for Figure 3 at 24h of treatment. Ordinary one-way ANOVA with post-hoc Tukey test. Table S9. Data analysis results for Figure 3 at 48h of treatment. Ordinary one-way ANOVA with post-hoc Tukey test. Table S10. Data analysis results for Figure 3 at 72h of treatment. Ordinary one-way ANOVA with post-hoc Tukey test. Table S11. Data analysis results for Figure 3 for media treatment. Kluskal-Wallis test with post-hoc Dunn’s test. Table S12. Data analysis results for Figure 3 for 1 ng/ml IL-1β treatment. Kluskal-Wallis test with post-hoc Dunn’s test. Table S13. Data analysis results for Figure 3 for 10 ng/ml IL-1β treatment. Kluskal-Wallis test with post-hoc Dunn’s test. Table S14. Data analysis results for Figure 3 for 100 ng/ml IL-1β treatment. Kluskal-Wallis test with post-hoc Dunn’s test. Table S15. Data for Figure S2A at 6h of treatment. Table S16. Data analysis results for Figure S2A at 6h of treatment. Ordinary one-way ANOVA with post-hoc Tukey test. Table S17. Data for Figure S2B at 24h of treatment. Table S18. Data analysis results for Figure S2B at 24h of treatment. Ordinary one-way ANOVA with post-hoc Tukey test. Table S19. Data for Figure S2C at 48h of treatment. Table S20. Data analysis results for Figure S2C at 48h of treatment. Ordinary one-way ANOVA with post-hoc Tukey test. Table S21. Data for Figure S2D at 72h of treatment. Table S22. Data analysis results for Figure S2D at 72h of treatment. Ordinary one-way ANOVA with post-hoc Tukey test. Table S23. D [file pone.0316110.s004.pdf]

Table S1. Data for Figure 2 MTT assay.

Media		1 ng		10 ng		100 ng	
Mean	SEM	Mean	SEM	Mean	SEM	Mean	SEM
100	133.8941	96.12904	135.335	87.74194	125.592	88.70968	124.1668
100	135.9025	97.07317	135.8073	95.12195	130.7565	87.47968	124.5277
100	150.006	92.33227	142.5023	99.04153	146.0338	91.05431	133.0654
100	145.3294	101.0363	147.0596	94.30052	138.7558	105.5268	149.9947

Table S2. Data analysis results for Figure 2. Ordinary one-way ANOVA with post-hoc Tukey test.

Tukey's multiple comparisons test	Mean Diff.	95.00% CI of diff.	Below threshold?	Summary	Adjusted P Value	
Media vs. 1 ng	3.357	-7.376 to 14.09	No	ns	0.7904	A-B
Media vs. 10 ng	5.949	-4.785 to 16.68	No	ns	0.3917	A-C
Media vs. 100 ng	6.807	-3.926 to 17.54	No	ns	0.2852	A-D
1 ng vs. 10 ng	2.591	-8.142 to 13.32	No	ns	0.8886	B-C
1 ng vs. 100 ng	3.45	-7.283 to 14.18	No	ns	0.7769	B-D
10 ng vs. 100 ng	0.8589	-9.875 to 11.59	No	ns	0.995	C-D

Table S3. Data for Figure S1A THP-1 monocytes control.

	IL-6 (pg/mL)	
	Rep #1	Rep #2
Media	1.52186	1.179068
LPS	19.49796	21.29616

Table S4. Data for Figure S1B Standard curve example.

IL-6 pg/mL	Optical Density	
600	3.015	2.899
300	2.258	N/A
150	N/A	1.319
75	0.821	0.757
37.5	0.455	0.409
18.75	0.231	0.224
9.375	0.14	0.116
4.6875	0.089	0.067
0	0.019	0.014

Table S5. Data for Figure S1C Sample dilution optimization.

Dilution	1/10	1/20	1/40	1/80	1/160	1/320	1/640
IL-6 ng/mL	N/A	17.32919	25.34565	30.0071	37.48308	39.40285	40.11022

Table S6. Data for Figure 3 IL-1β stimulation.

Hours	Media		1 ng/mL		10 ng/mL		100 ng/mL	
	Rep #1	Rep #2	Rep #1	Rep #2	Rep #1	Rep #2	Rep #1	Rep #2
**6**	2.324767	2.373434	7.442191	5.868991	7.873937	5.657696	8.05241	5.330117
**24**	4.606785	3.206264	21.91266	10.80058	23.1007	17.42569	23.78121	20.32377
**48**	7.56909	5.887439	49.80691	26.7195	45.20332	39.15314	45.65532	46.00385
**72**	10.94255	6.787305	45.20332	25.29672	68.03488	43.18261	71.28402	43.05597

Table S7. Data analysis results for Figure 3 at 6h of treatment. Ordinary one-way ANOVA with post-hoc Tukey test.

Tukey's multiple comparisons test	Mean Diff.	95.00% CI of diff.	Below threshold?	Summary	Adjusted P Value	
Media vs. 1ng	-4306	-9843 to 1230	No	ns	0.1071	A-B
Media vs. 10ng	-4417	-9954 to 1120	No	ns	0.0996	A-C
Media vs. 100ng	-4342	-9879 to 1195	No	ns	0.1046	A-D
1ng vs. 10ng	-110.2	-5647 to 5427	No	ns	0.9998	B-C
1ng vs. 100ng	-35.67	-5573 to 5501	No	ns	>0.9999	B-D
10ng vs. 100ng	74.55	-5462 to 5612	No	ns	>0.9999	C-D

Table S8. Data analysis results for Figure 3 at 24h of treatment. Ordinary one-way ANOVA with post-hoc Tukey test.

Tukey's multiple comparisons test	Mean Diff.	95.00% CI of diff.	Below threshold?	Summary	Adjusted P Value	
Media vs. 1ng	-12450	-31194 to 6293	No	ns	0.1638	A-B
Media vs. 10ng	-16357	-35100 to 2387	No	ns	0.0764	A-C
Media vs. 100ng	-18146	-36890 to 597.6	No	ns	0.0554	A-D
1ng vs. 10ng	-3907	-22650 to 14837	No	ns	0.8305	B-C
1ng vs. 100ng	-5696	-24439 to 13048	No	ns	0.6395	B-D
10ng vs. 100ng	-1789	-20533 to 16954	No	ns	0.9775	C-D

Table S9. Data analysis results for Figure 3 at 48h of treatment. Ordinary one-way ANOVA with post-hoc Tukey test.

Tukey's multiple comparisons test	Mean Diff.	95.00% CI of diff.	Below threshold?	Summary	Adjusted P Value	
Media vs. 1ng	-31535	-65975 to 2905	No	ns	0.066	A-B
Media vs. 10ng	-35450	-69890 to -1010	Yes	*	0.0455	A-C
Media vs. 100ng	-39101	-73541 to -4662	Yes	*	0.0329	A-D
1ng vs. 10ng	-3915	-38355 to 30525	No	ns	0.9635	B-C
1ng vs. 100ng	-7566	-42006 to 26873	No	ns	0.8094	B-D
10ng vs. 100ng	-3651	-38091 to 30788	No	ns	0.9699	C-D

Table S10. Data analysis results for Figure 3 at 72h of treatment. Ordinary one-way ANOVA with post-hoc Tukey test.

Tukey's multiple comparisons test	Mean Diff.	95.00% CI of diff.	Below threshold?	Summary	Adjusted P Value	
Media vs. 1ng	-26385	-87921 to 35151	No	ns	0.4101	A-B
Media vs. 10ng	-46744	-108280 to 14792	No	ns	0.1144	A-C
Media vs. 100ng	-48305	-109841 to 13231	No	ns	0.1043	A-D
1ng vs. 10ng	-20359	-81895 to 41177	No	ns	0.5852	B-C
1ng vs. 100ng	-21920	-83456 to 39616	No	ns	0.536	B-D
10ng vs. 100ng	-1561	-63097 to 59975	No	ns	0.9995	C-D

Table S11. Data analysis results for Figure 3 for media treatment. Kluskal-Wallis test with post-hoc Dunn’s test.

Dunn's multiple comparisons test	Mean rank diff.	Significant?	Summary	Adjusted P Value	
6h vs. 24h	-2	No	ns	>0.9999	A-B
6h vs. 48h	-4.5	No	ns	0.3972	A-C
6h vs. 72h	-5.5	No	ns	0.1485	A-D
24h vs. 48h	-2.5	No	ns	>0.9999	B-C
24h vs. 72h	-3.5	No	ns	0.9183	B-D
48h vs. 72h	-1	No	ns	>0.9999	C-D

Table S12. Data analysis results for Figure 3 for 1 ng/ml IL-1β treatment. Kluskal-Wallis test with post-hoc Dunn’s test.

Dunn's multiple comparisons test	Mean rank diff.	Significant?	Summary	Adjusted P Value	
6h vs. 24h	-2	No	ns	>0.9999	A-B
6h vs. 48h	-5.5	No	ns	0.1485	A-C
6h vs. 72h	-4.5	No	ns	0.3972	A-D
24h vs. 48h	-3.5	No	ns	0.9183	B-C
24h vs. 72h	-2.5	No	ns	>0.9999	B-D
48h vs. 72h	1	No	ns	>0.9999	C-D

Table S13. Data analysis results for Figure 3 for 10 ng/ml IL-1β treatment. Kluskal-Wallis test with post-hoc Dunn's test.

Dunn's multiple comparisons test	Mean rank diff.	Significant?	Summary	Adjusted P Value	
6h vs. 24h	-2	No	ns	>0.9999	A-B
6h vs. 48h	-4.5	No	ns	0.3972	A-C
6h vs. 72h	-5.5	No	ns	0.1485	A-D
24h vs. 48h	-2.5	No	ns	>0.9999	B-C
24h vs. 72h	-3.5	No	ns	0.9183	B-D
48h vs. 72h	-1	No	ns	>0.9999	C-D

Table S14. Data analysis results for Figure 3 for 100 ng/ml IL-1β treatment. Kluskal-Wallis test with post-hoc Dunn's test.

Dunn's multiple comparisons test	Mean rank diff.	Significant?	Summary	Adjusted P Value	
6h vs. 24h	-2	No	ns	>0.9999	A-B
6h vs. 48h	-5	No	ns	0.2474	A-C
6h vs. 72h	-5	No	ns	0.2474	A-D
24h vs. 48h	-3	No	ns	>0.9999	B-C
24h vs. 72h	-3	No	ns	>0.9999	B-D
48h vs. 72h	0	No	ns	>0.9999	C-D

Table S15. Data for Figure S2A at 6h of treatment.

	Media	1ng ng/mL	10ng ng/mL	100ng ng/mL
Rep #1	2324.767	7442.191	7873.937	8052.41
Rep #2	2373.434	5868.991	5657.696	5330.117

Table S16. Data analysis results for Figure S2A at 6h of treatment. Ordinary one-way ANOVA with post-hoc Tukey test.

Tukey's multiple comparisons test	Mean Diff.	95.00% CI of diff.	Below threshold?	Summary	Adjusted P Value	
Media vs. 1ng	-4306	-9843 to 1230	No	ns	0.1071	A-B
Media vs. 10ng	-4417	-9954 to 1120	No	ns	0.0996	A-C
Media vs. 100ng	-4342	-9879 to 1195	No	ns	0.1046	A-D
1ng vs. 10ng	-110.2	-5647 to 5427	No	ns	0.9998	B-C
1ng vs. 100ng	-35.67	-5573 to 5501	No	ns	>0.9999	B-D
10ng vs. 100ng	74.55	-5462 to 5612	No	ns	>0.9999	C-D

Table S17. Data for Figure S2B at 24h of treatment.

	Media	1ng ng/mL	10ng ng/mL	100ng ng/mL
Rep #1	4606.785	21912.66	23100.7	23781.21
Rep #2	3206.264	10800.58	17425.69	20323.77

Table S18. Data analysis results for Figure S2B at 24h of treatment. Ordinary one-way ANOVA with post-hoc Tukey test.

Tukey's multiple comparisons test	Mean Diff.	95.00% CI of diff.	Below threshold?	Summary	Adjusted P Value	
Media vs. 1ng	-12450	-31194 to 6293	No	ns	0.1638	A-B
Media vs. 10ng	-16357	-35100 to 2387	No	ns	0.0764	A-C
Media vs. 100ng	-18146	-36890 to 597.6	No	ns	0.0554	A-D
1ng vs. 10ng	-3907	-22650 to 14837	No	ns	0.8305	B-C
1ng vs. 100ng	-5696	-24439 to 13048	No	ns	0.6395	B-D
10ng vs. 100ng	-1789	-20533 to 16954	No	ns	0.9775	C-D

Table S19. Data for Figure S2C at 48h of treatment.

	Media	1ng ng/mL	10ng ng/mL	100ng ng/mL
Rep #1	7569.09	49806.91	45203.32	45655.32
Rep #2	5887.439	26719.5	39153.14	46003.85

Table S20. Data analysis results for Figure S2C at 48h of treatment. Ordinary one-way ANOVA with post-hoc Tukey test.

Tukey's multiple comparisons test	Mean Diff.	95.00% CI of diff.	Below threshold?	Summary	Adjusted P Value	
Media vs. 1ng	-31535	-65975 to 2905	No	ns	0.066	A-B
Media vs. 10ng	-35450	-69890 to -1010	Yes	*	0.0455	A-C
Media vs. 100ng	-39101	-73541 to -4662	Yes	*	0.0329	A-D
1ng vs. 10ng	-3915	-38355 to 30525	No	ns	0.9635	B-C
1ng vs. 100ng	-7566	-42006 to 26873	No	ns	0.8094	B-D
10ng vs. 100ng	-3651	-38091 to 30788	No	ns	0.9699	C-D

Table S21. Data for Figure S2D at 72h of treatment.

	Media	1ng ng/mL	10ng ng/mL	100ng ng/mL
Rep #1	10942.55	45203.32	68034.88	71284.02
Rep #2	6787.305	25296.72	43182.61	43055.97

Table S22. Data analysis results for Figure S2D at 72h of treatment. Ordinary one-way ANOVA with post-hoc Tukey test.

Tukey's multiple comparisons test	Mean Diff.	95.00% CI of diff.	Below threshold?	Summary	Adjusted P Value	
Media vs. 1ng	-26385	-87921 to 35151	No	ns	0.4101	A-B
Media vs. 10ng	-46744	-108280 to 14792	No	ns	0.1144	A-C
Media vs. 100ng	-48305	-109841 to 13231	No	ns	0.1043	A-D
1ng vs. 10ng	-20359	-81895 to 41177	No	ns	0.5852	B-C
1ng vs. 100ng	-21920	-83456 to 39616	No	ns	0.536	B-D
10ng vs. 100ng	-1561	-63097 to 59975	No	ns	0.9995	C-D

Table S23. Data for Figure S3A for media treatment.

	6h	24h	48h	72h
Rep #1	2324.767	4606.785	7569.09	10942.55
Rep #2	2373.434	3206.264	5887.439	6787.305

Table S24. Data analysis results for Figure S3A for media treatment. Kluskal-Wallis test with post-hoc Dunn's test.

Dunn's multiple comparisons test	Mean rank diff.	Significant?	Summary	Adjusted P Value	
6h vs. 24h	-2	No	ns	>0.9999	A-B
6h vs. 48h	-4.5	No	ns	0.3972	A-C
6h vs. 72h	-5.5	No	ns	0.1485	A-D
24h vs. 48h	-2.5	No	ns	>0.9999	B-C
24h vs. 72h	-3.5	No	ns	0.9183	B-D
48h vs. 72h	-1	No	ns	>0.9999	C-D

Table S25. Data for Figure S3B for 1 ng/ml IL-1β treatment.

	6h	24h	48h	72h
Rep #1	7442.191	21912.66	49806.91	45203.32
Rep #2	5868.991	10800.58	26719.5	25296.72

Table S26. Data analysis results for Figure S3B for 1 ng/ml IL-1β treatment. Kluskal-Wallis test with post-hoc Dunn's test.

Dunn's multiple comparisons test	Mean rank diff.	Significant?	Summary	Adjusted P Value	
6h vs. 24h	-2	No	ns	>0.9999	A-B
6h vs. 48h	-5.5	No	ns	0.1485	A-C
6h vs. 72h	-4.5	No	ns	0.3972	A-D
24h vs. 48h	-3.5	No	ns	0.9183	B-C
24h vs. 72h	-2.5	No	ns	>0.9999	B-D
48h vs. 72h	1	No	ns	>0.9999	C-D

Table S27. Data for Figure S3C for 10 ng/ml IL-1β treatment.

	6h	24h	48h	72h
Rep #1	7873.937	23100.7	45203.32	68034.88
Rep #2	5657.696	17425.69	39153.14	43182.61

Table S28. Data analysis results for Figure S3C for 10 ng/ml IL-1β treatment. Kluskal-Wallis test with post-hoc Dunn's test.

Dunn's multiple omparisons test	Mean rank diff.	Significant?	Summary	Adjusted P Value	
6h vs. 24h	-2	No	ns	>0.9999	A-B
6h vs. 48h	-4.5	No	ns	0.3972	A-C
6h vs. 72h	-5.5	No	ns	0.1485	A-D
24h vs. 48h	-2.5	No	ns	>0.9999	B-C
24h vs. 72h	-3.5	No	ns	0.9183	B-D
48h vs. 72h	-1	No	ns	>0.9999	C-D

Table S29. Data for Figure S3D for 100 ng/ml IL-1β treatment.

	6h	24h	48h	72h
Rep #1	8052.41	23781.21	45655.32	71284.02
Rep #2	5330.117	20323.77	46003.85	43055.97

Table S30. Data analysis results for Figure S3D for 100 ng/ml IL-1β treatment. Kluskal-Wallis test with post-hoc Dunn's test.

Dunn's multiple comparisons test	Mean rank diff.	Significant?	Summary	Adjusted P Value	
6h vs. 24h	-2	No	ns	>0.9999	A-B
6h vs. 48h	-5	No	ns	0.2474	A-C
6h vs. 72h	-5	No	ns	0.2474	A-D
24h vs. 48h	-3	No	ns	>0.9999	B-C
24h vs. 72h	-3	No	ns	>0.9999	B-D
48h vs. 72h	0	No	ns	>0.9999	C-D

Table S31. Data for Figure 4 MTT assay.

	Media	0.001 nM canakinumab	0.01 nM canakinumab	0.1 nM canakinumab	1 nM canakinumab	10 nM canakinumab	100 nM canakinumab	1 nM HS canakinumab	10 HS canakinumab	100 HS canakinumab
Rep #1	109.857	120.052	127.3342	121.9246	131.4954	125.0455	139.4018	123.173	126.502	131.7035
Rep #2	96.74901	104.2393	111.9376	117.7633	123.7971	123.173	138.7776	125.0455	122.5488	128.9987
Rep #3	98.20551	102.1586	114.2263	124.8375	118.1795	118.3875	130.6632	128.1664	115.2666	133.7841
Rep #4	105.4876	109.4408	123.173	124.2133	130.2471	131.9116	125.8778	133.1599	122.5488	130.2471
Rep #5	101.7425	97.58131	114.8505	122.1326	124.8375	129.6229	117.7633	N/A	N/A	133.5761
Rep #6	103.407	103.8231	120.4681	120.052	110.065	118.1795	118.8036	N/A	N/A	131.7035
Rep #7	103.407	107.3602	117.7633	116.723	116.0988	121.7165	126.502	N/A	N/A	134.6164
Rep #8	81.14431	102.9909	116.3069	123.5891	136.0728	146.2679	138.5696	N/A	N/A	146.4759

Table S32. Data analysis results for Figure 4. Kluskal-Wallis test with post-hoc Dunn's test.

Dunn's multiple comparisons test	Mean rank diff.	Significant?	Summary	Adjusted P Value
Media vs. 0.001	-4.938	No	ns	>0.9999
Media vs. 0.01	-21.25	No	ns	0.3803
Media vs. 0.1	-28.25	No	ns	0.0624
Media vs. 1	-34.94	Yes	**	0.0076
Media vs. 10	-38.75	Yes	**	0.0019
Media vs. 100	-45.44	Yes	***	0.0001
Media vs. 1 HS	-43.88	Yes	**	0.0056
Media vs. 10 HS	-29.38	No	ns	0.197
Media vs. 100 HS	-55.31	Yes	****	<0.0001

Note: The significant values indicate higher proliferation rate, not toxicity.

Table S33. Data for Figure 5A for pre-treatment with canakinumab.

Media	IL-1β	HS	1000nM canakinumab	500nM canakinumab	250nM canakinumab	100nM canakinumab	10nM canakinumab	1nM canakinumab	0.1nM canakinumab	0.01nM canakinumab	0.001nM canakinumab
33.20402	104.0249	122.7503	20.94225	27.73574	40.11564	28.38973	23.67314	34.46224	46.02365	103.0357	94.03195
10.8947	111.5922	104.3799	20.4404	25.38432	33.8516	27.88927	36.64743	34.21758	88.84455	117.9225	250.9781
25.639	168.5322	109.6136	60.9832	50.5762	50.7418	51.8499	65.797	122.0975	140.9012	172.4811	170.4475
35.40039	176.7071	123.5604	46.75791	53.35745	52.2395	52.6858	64.1777	114.0783	141.8779	174.9455	176.9152

Table S34. Data analysis results for Figure 5A for pre-treatment with canakinumab. Ordinary one-way ANOVA with post-hoc Tukey test.

Tukey's multiple comparisons test	Mean Diff.	95.00% CI of diff.	Below threshold?	Summary	Adjusted P Value	
IL-1β vs. 1000nM	102.9	21.96 to 183.9	Yes	**	0.0041	B-D
IL-1β vs. 500nM	101	19.97 to 181.9	Yes	**	0.0052	B-E
IL-1β vs. 250nM	95.98	15.00 to 177.0	Yes	**	0.0094	B-F
IL-1β vs. 100nM	100	19.03 to 181.0	Yes	**	0.0058	B-G
IL-1β vs. 10nM	92.64	11.66 to 173.6	Yes	*	0.0139	B-H
IL-1β vs. 1nM	64	-16.98 to 145.0	No	ns	0.2401	B-I
IL-1β vs. 0.1nM	35.8	-45.18 to 116.8	No	ns	0.9177	B-J
IL-1β vs. 0.01nM	-1.882	-82.86 to 79.10	No	ns	>0.9999	B-K
IL-1β vs. 0.001nM	-32.88	-113.9 to 48.10	No	ns	0.9523	B-L

Table S35. Data for Figure 5A for co-incubation of canakinumab and IL-1β.

Media	IL-1β	HS	1000nM canakinumab	500nM canakinumab	250nM canakinumab	100nM canakinumab	10nM canakinumab	1nM canakinumab	0.1nM canakinumab	0.01nM canakinumab	0.001nM canakinumab
18.00244	98.5986	151.7943	23.67314	26.81929	23.08447	19.62125	24.82324	23.63625	104.5932	N/A	99.9704
33.00244	185.0349	89.09935	16.82235	18.7031	21.33828	27.58245	20.54774	16.27176	172.3413	N/A	134.0895
48.43818	167.6305	137.2124	49.4764	47.78575	46.32701	55.72925	54.1444	52.5741	89.38515	135.0468	173.8128
47.67726	178.4821	118.6446	49.03847	61.21785	45.36155	50.79705	55.50195	56.6987	88.22315	213.8952	176.6031

Table S36. Data analysis results for Figure 5A for co-incubation of canakinumab and IL-1β. Ordinary one-way ANOVA with post-hoc Tukey test.

Tukey's multiple comparisons test	Mean Diff.	95.00% CI of diff.	Below threshold?	Summary	Adjusted P Value	
IL-1β vs. 1000nM	122.7	55.25 to 190.1	Yes	****	<0.0001	B-D
IL-1β vs. 500nM	118.8	51.37 to 186.2	Yes	****	<0.0001	B-E
IL-1β vs. 250nM	123.4	55.98 to 190.8	Yes	****	<0.0001	B-F
IL-1β vs. 100nM	119	51.57 to 186.4	Yes	****	<0.0001	B-G
IL-1β vs. 10nM	118.7	51.25 to 186.1	Yes	****	<0.0001	B-H
IL-1β vs. 1nM	120.1	52.71 to 187.6	Yes	****	<0.0001	B-I
IL-1β vs. 0.1nM	43.8	-23.63 to 111.2	No	ns	0.5111	B-J
IL-1β vs. 0.01nM	-17.03	-99.62 to 65.55	No	ns	0.9998	B-K
IL-1β vs. 0.001nM	11.32	-56.12 to 78.75	No	ns	>0.9999	B-L

Table S37. Data used for Figure 5B for pre-treatment with canakinumab. Analysis of Inhibition dose-response using a non-linear regression model to calculate IC_{50}, per treatment time point.

	IL-6 Concentration (ng/ml)			
Canakinumab Concentration (nM)	Rep #1	Rep #2	Rep #3	Rep #4
1000	17.05104	16.64244	43.43893	33.30612
500	19.91723	18.22866	31.2348	32.95245
250	28.69057	24.21055	31.44712	32.37532
100	18.85249	18.52015	27.74844	28.19579
10	16.08354	24.89828	37.72985	36.8013
1	24.54779	24.37351	66.34888	61.99115
0.1	36.46272	70.38804	85.81629	86.41116
0.01	73.69075	84.33774	107.651	109.1891
0.001	70.9918	N/A	93.77784	97.33625
0	80.25299	86.09098	114.0616	119.5944

Table S38. Data used for Figure 5B for co-incubation of canakinumab and IL-1β. Analysis of Inhibition dose-response using a non-linear regression model to calculate IC_{50}, per treatment time point.

	IL-6 Concentration (ng/ml)			
**Canakinumab Concentration (nM)**	**Rep #1**	**Rep #2**	**Rep #3**	**Rep #4**
1000	28.23276	27.98286	24.53719	N/A
500	24.00413	30.75146	28.38233	19.79312
250	23.19426	22.71089	22.53556	20.8309
100	26.33098	24.00061	19.79421	27.82558
10	26.40804	27.07016	22.62488	18.72801
1	27.60256	29.76807	24.16735	16.63738
0.1	47.42357	46.80706	112.2617	N/A
0.01	70.41306	111.5245	N/A	N/A
0.001	91.25554	92.72047	119.1506	N/A

Table S39. Data for Figure 6 MTT assay.

Media	IL-1β 10 ng/ml	Canakinumab 10 nM	IL-1β 10 ng/ml + Canakinumab 10 nM
94.41599	97.31538	94.63902	109.8051
115.9012	103.1142	88.54287	100.6608
106.9057	106.2366	95.82851	99.39699
94.9364	113.3384	90.27882	111.0023
95.23377	122.7581	94.57423	104.8983
105.8649	125.5463	88.54559	104.2954
94.63902	N/A	N/A	N/A
100.1404	N/A	N/A	N/A
91.96266	N/A	N/A	N/A
121.7784	N/A	N/A	N/A
104.8983	N/A	N/A	N/A
104.5215	N/A	N/A	N/A
80.55765	N/A	N/A	N/A
94.04672	N/A	N/A	N/A
90.5049	N/A	N/A	N/A
103.165	N/A	N/A	N/A
107.0083	N/A	N/A	N/A
93.51922	N/A	N/A	N/A

Table S40. Data analysis results for Figure 6. Ordinary one-way ANOVA with post-hoc Dunnett's multiple comparisons test.

Dunnett's multiple comparisons test	Mean Diff.	95.00% CI of diff.	Below threshold?	Summary	Adjusted P Value	A-?
MEDIA vs. Canakinumab 10 nM	7.932	-2.333 to 18.20	No	ns	0.1662	B
MEDIA vs. IL-1β 10 ng/ml + Canakinumab 10 nM	-5.01	-15.28 to 5.255	No	ns	0.5233	C
MEDIA vs. IL-1β 10 ng/ml	-11.38	-21.65 to -1.120	Yes	*	0.0263	D

Note: The significant values indicate higher proliferation rate, not toxicity.

Table S41. Data for Figure 7A myoblasts.

Media	IL-1β 10 ng/ml	Canakinumab 10 nM	IL-1β 10 ng/ml + Canakinumab 10 nM
5.321503	69.39121	5.286203	32.31844
5.216419	63.08957	5.321503	31.75319
5.447194	64.27	5.321503	29.54724
5.357075	49.50166	5.321503	29.54724
5.357075	63.72376	5.321503	23.09045
5.392919	56.16312	5.339255	25.17944

Table S42. Data analysis results for Figure 7A. Ordinary one-way ANOVA with post-hoc Tukey test.

Tukey's multiple comparisons test	Mean Diff.	95.00% CI of diff.	Below threshold?	Summary	Adjusted P Value	
Media vs. IL-1β 10 ng/ml	-55.67	-62.10 to -49.25	Yes	****	<0.0001	A-B
Media vs. Canakinumab 10 nM	0.03012	-6.395 to 6.455	No	ns	>0.9999	A-C
Media vs. IL-1β 10 ng/ml + Canakinumab 10 nM	-23.22	-29.65 to -16.80	Yes	****	<0.0001	A-D
IL-1β 10 ng/ml vs. Canakinumab 10 nM	55.7	49.28 to 62.13	Yes	****	<0.0001	B-C
IL-1β 10 ng/ml vs. IL-1β 10 ng/ml + Canakinumab 10 nM	32.45	26.03 to 38.88	Yes	****	<0.0001	B-D
Canakinumab 10 nM vs. IL-1β 10 ng/ml + Canakinumab 10 nM	-23.25	-29.68 to -16.83	Yes	****	<0.0001	C-D

Table S43. Data for Figure 7B myotubes.

Media	IL-1β 10 ng/ml	Canakinumab 10 nM	IL-1β 10 ng/ml + Canakinumab 10 nM
6.208424	80.96917	6.292343	48.00365
6.355995	93.47735	6.441817	52.90009
6.229302	84.19272	6.271261	58.56954
6.229302	97.28665	6.313492	43.73117
6.229302	100.9436	6.441817	34.5035
6.229302	98.64986	6.506897	44.85469

Table S44. Data analysis results for Figure 7B. Ordinary one-way ANOVA with post-hoc Tukey test.

Tukey's multiple comparisons test	Mean Diff.	95.00% CI of diff.	Below threshold?	Summary	Adjusted P Value	
Media vs. IL-1β 10 ng/ml	-86.34	-95.73 to -76.94	Yes	****	<0.0001	A-B
Media vs. Canakinumab 10 nM	-0.131	-9.526 to 9.264	No	ns	>0.9999	A-C
Media vs. IL-1β 10 ng/ml + Canakinumab 10 nM	-40.85	-50.24 to -31.45	Yes	****	<0.0001	A-D
IL-1β 10 ng/ml vs. Canakinumab 10 nM	86.21	76.81 to 95.60	Yes	****	<0.0001	B-C
IL-1β 10 ng/ml vs. IL-1β 10 ng/ml + Canakinumab 10 nM	45.49	36.10 to 54.89	Yes	****	<0.0001	B-D
Canakinumab 10 nM vs. IL-1β 10 ng/ml + Canakinumab 10 nM	-40.72	-50.11 to -31.32	Yes	****	<0.0001	C-D
